# Correlation between facet tropism and lumbar degenerative disease: a retrospective analysis

**DOI:** 10.1186/s12891-017-1849-x

**Published:** 2017-11-22

**Authors:** Tian Gao, Qi Lai, Song Zhou, Xuqiang Liu, Yuan Liu, Ping Zhan, Xiaolong Yu, Jun Xiao, Min Dai, Bin Zhang

**Affiliations:** Department of Orthopedics, Artificial Joints Engineering and Technology Research Center of Jiangxi Province, The First Affiliated Hospital of Nanchang University, 17 Yongwai Street, Nanchang, 330006 Jiangxi People’s Republic of China

**Keywords:** Facet tropism, Lumbar disc herniation, Degenerative lumbar spondylolisthesis, Degenerative lumbar scoliosis, Lumbar degeneration diseases

## Abstract

**Background:**

The aim of this study was to investigate the correlation between facet tropism and spinal degenerative diseases, such as degenerative lumbar spondylolisthesis, degenerative lumbar scoliosis, and lumbar disc herniation.

**Methods:**

This study retrospectively analysed clinical data from the Department of Orthopaedics at The First Affiliated Hospital of Nanchang University. Ninety-two patients were diagnosed with lumbar spondylolisthesis, 64 patients with degenerative scoliosis, and 86 patients with lumbar disc herniation between 1 October 2014 and 1 October 2016. All patients were diagnosed using 3.0 T magnetic resonance imaging and underwent conservative or operative treatment. Facet tropism was defined as greater than a ten degree between the facet joint angles on both sides.

**Results:**

For L3-L4 degenerative lumbar spondylolisthesis, one out of six cases had tropism compared to seven out of the 86 controls (*p* = 0.474). At the L4-L5 level, 17/50 cases had tropism compared to 4/42 cases in the control group (*p* = 0.013). At the L5-S1 level, 18/36 cases had tropism compared to 7/56 controls (*p* = 0.000). For degenerative lumbar scoliosis at the L1-L5 level, 83/256 cases had tropism as compared to 36/256 controls (*p *= 0.000). For L3-L4 lumbar disc herniation two out of eight cases had tropism compared to 14/78 controls (*p* = 0.625). At the L4-L5 level, 19/44 cases had tropism compared to four out of 42 controls (*p* = 0.001). At the L5-S1 level, 24/34 cases had tropism compared to 10/52 controls (*p* = 0.000).

**Conclusion:**

At the L4–5 and L5-S1 levels, facet tropism is associated with degenerative spondylolisthesis. In the degenerative lumbar scoliosis group, the number of case with facet tropism was significantly higher than that of the control group. Facet tropism was associated with lumbar disc herniation at the L4–5 and L5-S1 levels. Overall, in these three lumbar degenerative diseases, facet tropism is a common phenomenon.

**Electronic supplementary material:**

The online version of this article (10.1186/s12891-017-1849-x) contains supplementary material, which is available to authorized users.

## Background

Lumbar degenerative diseases, such as lumbar disc herniation, degenerative lumbar scoliosis and lumbar spondylolisthesis, are common diseases in the field of orthopaedic medicine. The causes of lumbar degenerative diseases are multifactorial, with intervertebral disc degeneration being a major contributor. However, facet tropism, defined as asymmetry between the left and right facet joints, is postulated as a possible cause of lumbar degenerative diseases, especially in severe cases.

Some scholars state that the structural asymmetry of the lumbar facet joint is related to diseases of the lumbar spine. *Brailsford* [1] defined facet tropism, in 1928, as asymmetry between the left and right vertebral facet joint angles with one joint having a more sagittal orientation than the other. *Masharawi* et al. [2] found asymmetry in the facet orientation to be a normal characteristic in most thoracic vertebrae, but not in the lumbar vertebrae. They suggest, however, that when asymmetry in facet orientation occurs in the lumbar vertebrae, it may be related to pathologic conditions. *Farfan and Sullivan* [3] first suggested the correlation between facet tropism and the development of lumbar disc herniation. *Cassidy* [4] reported that the correlation between facet asymmetry and the side of disc herniation (sagittal or coronal) is debatable. *Fujiwara A* [5] reported that a more sagittal facet joint orientation arises secondary to osteoarthritis remodelling or as the result of osteoarthritis and facet joint effusion. *Grogan* et al. [6], in their study with 21 cadavers, insisted that lumbar facet joint tropism did not accelerate facet joint degeneration.

Currently, authors hypothesize that facet tropism may affect lumbar degenerative disease. However, the correlation between facet tropism and degenerative lumbar spondylolisthesis, degenerative lumbar scoliosis, lumbar disc herniation and other diseases has rarely been reported and remains controversial.

Previous investigations [7, 8] into the aetiology of degenerative disease of the lumbar spine focused on the degeneration of intervertebral discs, and there is a paucity of literature on lumbar degenerative disease as it relates to the lumbar facet joints. We are currently evaluating the lumbar facet joints and lumbar facet biomechanics, to better understand the lumbar facet joint, and its correlation with disease.

We aimed to assess the correlation between facet tropism and three degenerative diseases of the lumbar spine using a large sample, and analysed the relationship between the lumbar facet joint and lumbar degenerative disease.

## Methods

### General information

#### Degenerative lumbar spondylolisthesis

**Fig. 1:**
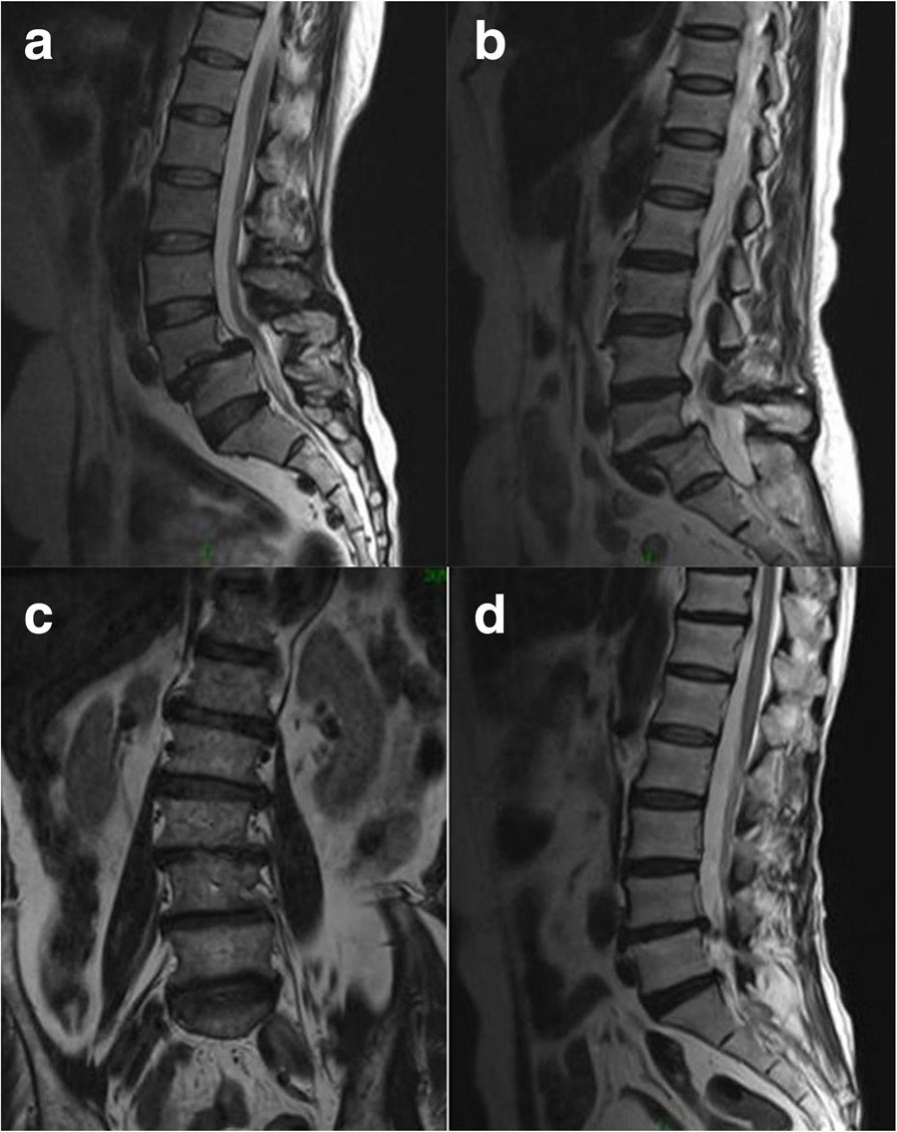
Lumbar degeneration diseases. The three representative degenerative diseases of the lumbar spine: **a**: L4 segmental degenerative lumbar spondylolisthesis; **b**: L5 segmental degenerative lumbar spondylolisthesis; **c**: Degenerative lumbar scoliosis; **d**: Lumbar disc herniation

The study was approved by the Ethics Committee of the First Affiliated Hospital of Nanchang University, China. We included patients from the Department of Orthopedics at our hospital from November 1, 2014 to November 1, 2016. There were 534 cases of lumbar spondylolisthesis (Fig. 1a, b) diagnosed by imaging and clinical manifestations, and 92 patients were eligible for this study, including 33 males and 59 females; Age range was 50–81 years old, with an average age of 68 years. A breakdown of the cohort characteristics is included in Table 1.

**Table 1:** Description of the Study Cohorts

Diagnosis	# Pts Diagnosed	# Pts Eligible	Avg Age	Age range	Females(%)
Lumbar Spondylolisthesis	534	92	68	50–81	59(64)
Lumbar Scoliosis	495	64	65	50–75	30(46)
Lumbar Disc Herniation	502	86	64	50–75	38(44)
Control Group	64	64	61	45–75	32(50)

Inclusion criteria were: (1) lumbar spondylolisthesis diagnosed by 3.0 T magnetic resonance imaging (MRI) and (2) L3, L4 or L5 single-segment lumbar spondylolisthesis. Exclusion criteria were: (1) MRI deficiency or multiple segments of lumbar spondylolisthesis and (2) diagnosis of lumbar disc herniation, transitional vertebral body, scoliosis, lumbar spine surgery, lumbar trauma or infection, lumbar facet joint infection or invasive tumour.

This study looked at specific levels of the spine and matched each level with the presence or absence of asymmetry. For example: If the patient’s L5 segment lumbar spondylolisthesis, then the L4–5, L3–4 segment was considered the control group.

#### Degenerative lumbar scoliosis

We included patients from the Department of Orthopedics at our hospital from November 1, 2014 to November 1, 2016. There were 495 cases of lumbar scoliosis (Fig. 1c) diagnosed by imaging and clinical manifestations, and 64 patients were eligible for this study, including 34 males and 30 females. Age range was 50–75 years old, with an average age of 65 years.

Inclusion criterion was lumbar scoliosis diagnosed by 3.0 T MRI. Exclusion criteria were: (1) lumbar scoliosis in the bone before maturity and (2) diagnosis of secondary scoliosis, lumbar disc herniation, lower lumbar vertebrae fracture, transitional vertebrae, lumbar spondylolisthesis, lumbar spine surgery, lumbar trauma or infection, lumbar facet joint infection, or invasive tumour.

We used a stratified sampling method to enrol a control group matched for age and gender. We selected 64 patients who were admitted to our hospital due to fracture of thoracic vertebra, and who had undergone X-ray and MRI. The control group included 32 males and 32 females with an age range of 45–75 years and a mean age of 61 years.

#### Lumbar disc herniation

We included patients from the Department of Orthopedics at our hospital from November 1, 2014 to November 1, 2016. There were 502 cases of lumbar disc herniation (Fig. 1d) diagnosed by imaging and clinical manifestations, and 86 patients were eligible for this study, including 48 male patients and 38 female patients. Age range was 50–75 years old, with an average age of 64 years.

Inclusion criteria were: (1) lumbar disc herniation diagnosed by 3.0 T MRI, (2) L3–4, L4–5 or L5-S1 single segment lumbar disc herniation and (3) lumbar intervertebral disc protrusion of 4 mm on MRI cross section of the lumbar spine. Exclusion criteria were: (1) MRI deficiency or multiple segments of lumbar disc herniation and (2) diagnosis of lumbar spondylolisthesis, lumbar vertebral fracture, transitional vertebrae, scoliosis, lumbar spine surgery, lumbar trauma or infection, lumbar facet joint infection, or invasive tumour.

This study looked at specific levels of the spine and matched each level with the presence or absence of asymmetry. For example: If the patient’s L5 segment of the disc is prominent, then the patient’s L4–5, L3–4 segment was considered the control group.

### Imaging examination and measurement methods

A total of 242 patients underwent MRI of the lumbar spine. We used 3.0 T MRI to obtain sagittal and traverse scans of the L1-S1 segments with a scanning layer thickness of 3 mm and a layer distance of 5 mm. Patients were evaluated in a supine position. If the patient was unable to remain still due to pain, pain relief was provided. T1 images of the left and right lumbar facet joints provide the clearest visualisation; therefore, we focused on T1 images to scan the lumbar facet joints. Although computed tomography has a higher resolution for bony structures, MRI is the best choice to evaluate the degree of degeneration and asymmetry of lumbar facet joints.

**Fig. 2:**
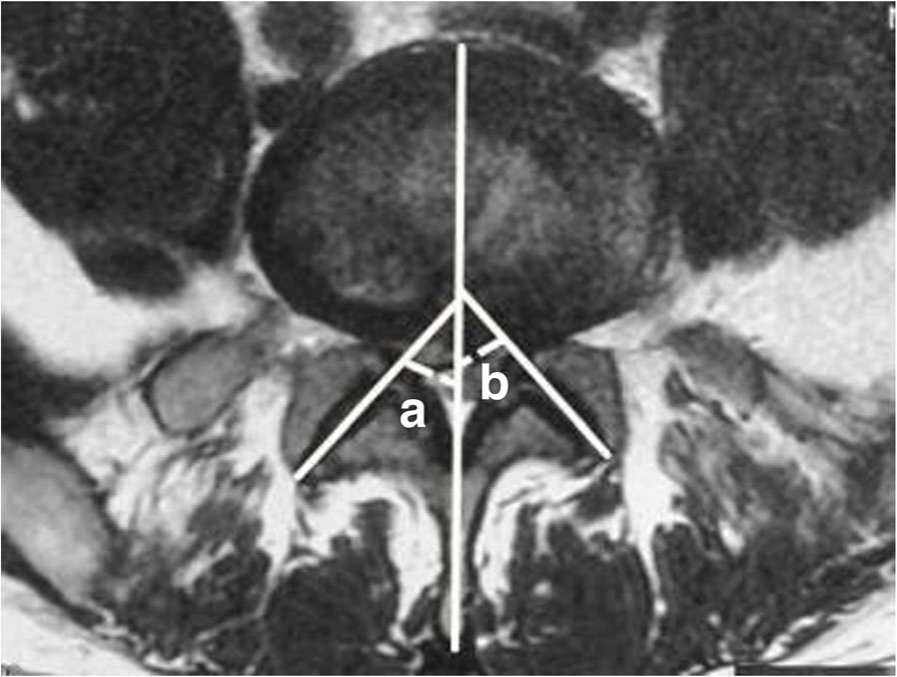
Facet joint measurement method. The difference in lumbar facet joint angle greater than or equal to 10° can be defined as lumbar facet joint asymmetry (a-b ≥ 10)

We used our hospital radiology department’s Haitai PACS clinical system to measure the cross-sectional angles. The imaging parameters of each patient were measured three times, and the average of the results was calculated. The angle of the lumbar facet joint is between the anterior and posterior ends of the articular surface of the superior articular process and the median sagittal line of the same vertebral body (Fig. 2a, b). A difference in the lumbar facet joint angle of greater than or equal to 10° was defined as lumbar facet joints asymmetry (a-b ≥ 10), as currently recognised by most scholars [9]. Since there is a range of error with the measurements, a 10° difference ensures that there is a true difference in symmetry. All cases were measured by two physicians.

### Statistical analysis

All data were analysed using SPSS 17.0 statistical software. The data is reported as a mean ± standard deviation. Standard T-tests were used to compare amongst the study groups; chi-square tests were used for the enumeration of data. A *p*-value of <0.05 was considered statistically significant.

## Results

In the 92 patients with lumbar spondylolisthesis, there was a statistically significant difference in facet tropism at the L4–5 and L5-S1 spinal levels, between the two groups (*p* < 0.05) (Table 2).In the degenerative lumbar scoliosis group, there were 83 facet joints with facet tropism, which accounted for 32.42% of the 256 facet joints. In the control group, asymmetry of the facet joints was present in 36; this difference was statistically significant (*p* < 0.05). In the single-segment comparison, asymmetry of the facet joints in the degenerative lumbar scoliosis group was significantly larger than asymmetry in the control group (*p* < 0.05) at the L2–3, L3–4, and L4–5 levels (Table 3).Amongst the 86 patients with lumbar herniation, statistical analysis showed that the difference was also statistically significant at the L4–5 and L5-S1 spinal levels (*p* < 0.05) (Table 4).

**Table 2:** Degenerative lumbar spondylolisthesis and control groups

spinal level	lumbar spondylolisthesis		facet tropism		χ^2^	*p*
			YES	NO		
L3–4	YES	6	1	5	0.514	0.474
	NO	86	7	79		
L4–5	YES	50	17	33	6.125	0.013
	NO	42	5	38		
L5-S1	YES	36	18	18	15.571	0.000
	NO	56	7	49

**Table 3:** Degenerative lumbar scoliosis and control groups

spinal level	group		facet tropism		χ^2^	*p*
			YES	NO		
L1–2	DLS	256	5	251	0.113	0.737
	CG	256	4	252		
L2–3	DLS	256	13	243	7.905	0.005
	CG	256	6	250		
L3–4	DLS	256	37	219	11.520	0.001
	CG	256	14	242		
L4–5	DLS	256	28	228	6.742	0.008
	CG	256	12	244		
L1–5	DLS	256	83	173	24.184	0.000
	CG	256	36	220		

DLS, degenerative lumbar scoliosis; CG, control group

**Table 4:** Disc herniation and control groups

spinal level	Disc herniation		facet tropism		χ^2^	*p*
			YES	NO		
L3–4	YES	8	2	6	0.238	0.625
	NO	78	14	64		
L4–5	YES	44	19	25	10.448	0.001
	NO	42	5	37		
L5-S1	YES	34	24	10	22.683	0.000
	NO	52	10	42		

## Discussion

As an important part of the three-joint complex of the spine, the lumbar facet joint has a far-reaching influence on the spine. Therefore, to further explore the correlation between facet tropism and lumbar degenerative disease, we selected three representative lumbar degenerative diseases and analysed their association with lumbar facet tropism.

### Correlation between facet tropism and degenerative lumbar spondylolisthesis

There is no consensus on the aetiology of degenerative lumbar spondylolisthesis; it is usually attributed to intervertebral disc degeneration and lumbar facet joint morphological abnormalities. *Sato* et al. [10] proposed that the lumbar spine, with a small joint, carries a risk for individual degenerative spondylolisthesis. *Boden* [11] measured the facet joint angle of 140 patients, using magnetic resonance imaging, and found that both the left and the right facet joints were more sagittally oriented in patients who had degenerative spondylolisthesis. He proposed that facet tropism was associated with degenerative lumbar spondylolisthesis. *Cinotti* [12] studied facet tropism with CT scans in 54 subjects (27 patients with and 27 without degenerative spondylolisthesis) to determine if there was an association between facet tropism and degenerative spondylolisthesis. Their results showed that facet joint orientation at the level of the spondylolisthesis, as well as levels above and below, was significantly different in patients with spondylolisthesis compared to the controls. An inverse linear correlation was found between the sagittal orientation of facet joints and the mobility of the slipped vertebra. In this study, we compared the number of asymmetric joints in degenerative spondylolisthesis and found that there was a significant correlation between lumbar facet joint asymmetry and spondylolisthesis at the L4-L5 and L5-S1 spinal levels. Therefore, this study provides new evidence of the correlation between the facet joint and lumbar spondylolisthesis, and provides the basis to study the mechanism of facet tropism and spondylolisthesis.

**Fig. 3:**
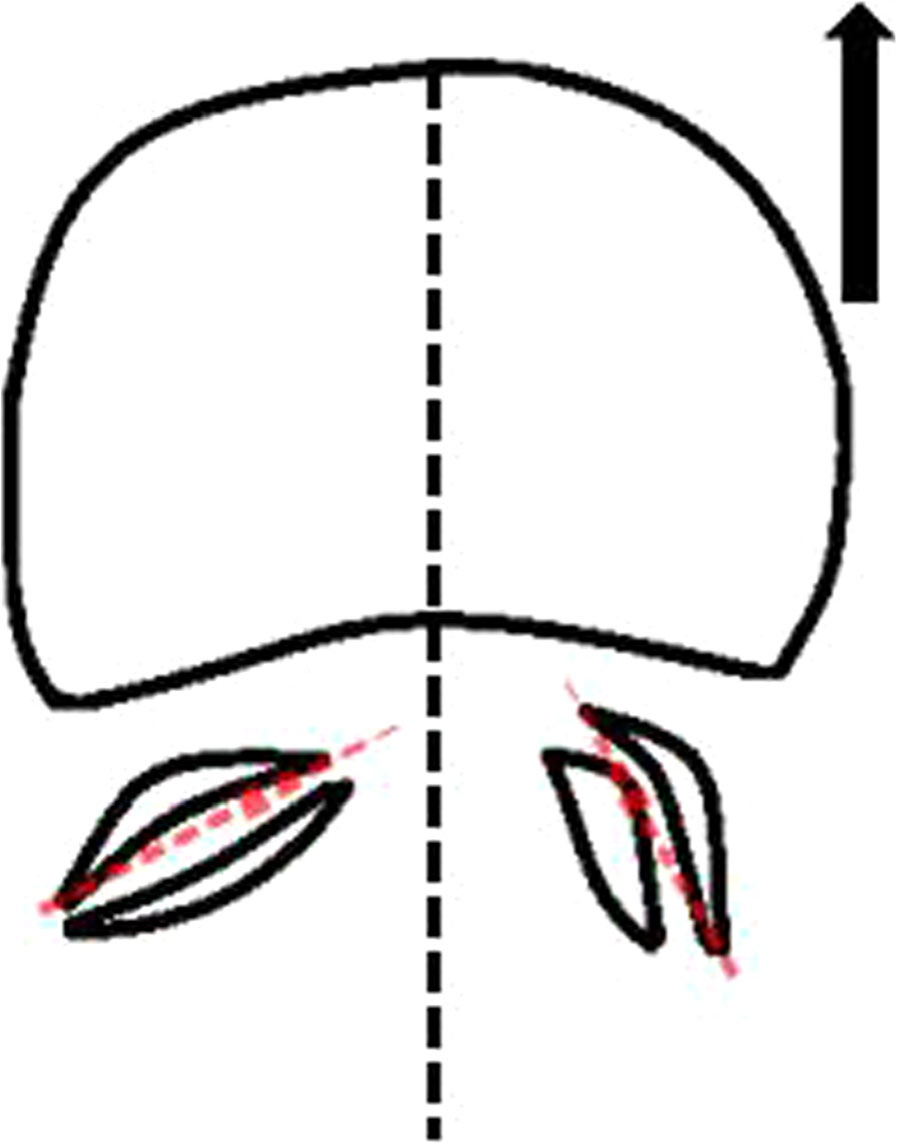
Schematic diagram of lumbar spondylolisthesis. With lumbar flexion, the vertebral body on the sagittal lateral facet joint and vertebral arch root angle side will more easily slide forward (as shown with the black arrow)

To explore the pathogenesis of degenerative lumbar spondylolisthesis, we hypothesise that facet tropism may be one of the initiating factors that may cause disease. Lumbar facet joint orientation allows the lumbar spine to complete a wide range of activities. If the vertebral facet joint angles are asymmetric, the posterior column may have mechanical deficits on the sagittal facet joint angle and the vertebral arch root angle. With lumbar flexion, the vertebral body, on the sagittal lateral facet joint and vertebral arch root angle side, will slide forward more easily (Fig. 3).

### Correlation between facet tropism and degenerative lumbar scoliosis

Degenerative lumbar scoliosis is a special type of scoliosis due to intervertebral disc and facet joint degeneration, which represents a new lateral curvature that develops in adulthood. This study was designed to investigate the correlation between the lumbar facet joints and degenerative lumbar scoliosis. The number of symmetric facet joints in the degenerative lumbar scoliosis group was significantly higher than in the control group, and the difference between patients and controls was statistically significant at the L2–3, L3–4 and L4–5 levels.

There is no definitive conclusion that degenerative lumbar scoliosis is caused by the degeneration of the intervertebral disc or the small joints, or by the interaction between intervertebral disc and facet tropism. *Kirkaldy-Willis* et al. [13] concluded that the intervertebral discs and the facet joints are interrelated and interact with each other at the three stages of spinal degeneration, and that any joint abnormality will affect the normal function of the other two joints. *Videman* et al. [14] showed that about 20% of small joint degenerative changes occur earlier than intervertebral disc degeneration. The results of this study show that there is a significant correlation between facet tropism and degenerative lumbar scoliosis,but the causal relationship is unclear. We hypothesise that there is a vicious cycle of facet joint degeneration, uneven mechanical distribution and spinal deformity, leading to the progressive development of degenerative scoliosis. Biomechanical studies may be able to clarify the mechanism between them. With the increasing prevalence of degenerative lumbar scoliosis, clinicians are increasingly concerned about these problems. We believe that with further research, the pathogenesis of degenerative lumbar scoliosis will become increasingly clear, and its prevention and treatment will also be improved.

### Correlation between facet tropism and lumbar disc herniation

Currently, there are few reports concerning the correlation between facet tropism and lumbar disc herniation, and the conclusions are not consistent. *Kalichman* [15, 16] and other studies report that the angle of the lumbar facet joint and lumbar disc herniation were significantly related to lumbar facet joint asymmetry and that more severe asymmetry was more likely to cause lumbar disc herniation. In contrast, *Badgley and Vanharanta* [17, 18] and other scholars believe that the angle of the lumbar spine and disc herniation do not correlate, and that lumbar facet joint asymmetry is a congenital structural manifestation, which is not due to age or degeneration. In this study, we compared the number of asymmetric joints in degenerative lumbar disc herniation and found that there was a significant correlation between lumbar facet joint asymmetry and lumbar disc herniation at the L4–5 and L5-S1 spinal levels.Therefore, this study provides new evidence for the correlation between the facet joint and lumbar disc herniation, and provides the basis to study the mechanism of facet tropism and lumbar herniation.

*Veres* [19] studied the mechanisms of facet tropism leading to disc herniation. He considered that both sides of the small joints and intervertebral disc together constitute the spinal three-joint complex. When the lumbar spine is flexed and twisted, if both joints are asymmetric, the stress of the three-joint complex is imbalanced. Resistance on the sides of the vertebral body is different, and the vertebral body will deviate from the original trajectory, thus pulling the rear of the intervertebral disc. With flexion of the lumbar spine, the nucleus pulposus will become prominent.

### Clinical significance

Our results show that there is a correlation between facet tropism and degenerative lumbar spondylolisthesis, degenerative scoliosis, and lumbar disc herniation. There are two possible clinical implications. First, doctors often focus only on disc degeneration. As such, if facet tropism were identified preoperatively, the joint could be corrected (for instance, with a bone graft fusion), so that the stability of the vertebral body can be reconstructed to reduce the possibility of recurrence and other complications. Also, patients with facet tropism should be regularly followed and examined. The authors believe that with further study of the lumbar facet joint, clinicians will be more attentive to the problem of facet tropism.

### Limitations

This study aimed to understand the correlation between the lumbar facet joints and degenerative lumbar diseases. There are some limitations to this study. In the analysis of patients with lumbar spondylolisthesis and lumbar disc herniation, we did not exclude patients that may have had one of the three pathologies present. We must clarify that the best study would be to identify these same patients before they developed any of the three diseases to see how many go on to develop disease. Furthermore, this study excluded multiple segment disc protrusions, which may have led to an underestimation of differences and the elimination of valuable data. Future studies should focus on the biomechanics of the lumbar facet joints, and further explore the correlation between facet tropism and lumbar degenerative disease.

## Conclusions

For the three lumbar degenerative diseases studied here, facet tropism was a common phenomenon with a significant correlation. Additionally, longitudinal studies are needed to understand the potential causal relationship between facet tropism and lumbar degenerative diseases.

## Additional files

Additional file 1: The dataset supporting the conclusions of this article. (XLSX 56 kb)

## Abbreviations

CG: Control Group
DLS: Degenerative lumbar scoliosis
DS: Degenerative spondylolisthesis
MRI: Magnetic resonance imaging
PACS: Picture Archiving and Communication Systems
